# The future of in silico trials and digital twins in medicine

**DOI:** 10.1093/pnasnexus/pgaf123

**Published:** 2025-04-18

**Authors:** Ehsan Samei

**Affiliations:** Department of Radiology and Center for Virtual Imaging Trials, Duke University, 2424 Erwin Road, Durham, NC 27710, USA

**Keywords:** in silico trials, digital twins, revolutionize medicine, qunatitative medicine, clinical trials, virtual clinical trials, regulatory science, prototyping

## Abstract

In silico trials and digital twins are emerging as transformative medical technologies, as they offer a unique way to design medical innovations, optimize their application, and evaluate their utility. Their use spans from individual care—appropriating the technology for personalized decision, to population care—presenting an alternative to design, supplement, or replace clinical trials. They effectually offer a new way to efficiently qualify, quantify, and personalize healthcare innovations in advance or in conjunction with clinical application. While much progress is underway to advance these technologies across diverse developments, realizing their full potential requires a cohesive goal to unify separate activities towards a common objective. Such a cohesive goal—moonshot—can be defined as forming and fostering a digital twin of every single human person, owned by the individual, progressively updated with new data, and used to deliver optimized care, technology assessment, and real-world evidence. The feasibility of such a vision builds upon a growing body of work in computational modeling, regulatory science, and digital healthcare. Bringing this vision to reality requires ownership and active engagement of all stakeholders to contribute diverse expertise and resources for transforming medicine and medical appropriation towards a more accurate, efficient, and quantitative future.

## The promise of digital twins in healthcare

In the last few years, in silico trials (ISTs) and digital twins (DTs) have emerged as transformative technologies in medicine, a move reflected not only in scientific circles, as underscored by major reports from the National Academies (1) and Food and Drug Administration (FDA) (2, 3), but also in the public periodicals (4). While definitions and interpretation can vary, consistent with the National Academies, a DT can be defined as “a set of virtual information constructs that mimics the structure, context, and behavior of a natural, engineered, or social system (or system-of-systems), is dynamically updated with data from its physical twin, has a predictive capability, and informs decisions that realize value” (1). Within this definition, a medical DT is then defined as “a viewable digital replica of a patient, organ, or biological system that contains multidimensional, patient-specific information, and informs decision” (5), and ISTs as systematic and robust computational analyses performed on or with DTs.

Several major initiatives, including the Avicenna Alliance, FDA, and European Medicines Agency regulatory frameworks, and the Virtual Physiological Human Initiative, have laid critical foundations for ISTs and DTs (1, 3, 6, 7). This emergence has been motivated by the severe need in biomedical sciences and in clinical practice to accelerate the evaluation of medical innovations and novel therapeutics, and to establish confidence in personalization of treatment plans. While clinical trials remain the gold standard for these evaluations, the cost, time, and ethics of these trials, in either human or preclinical animal forms, render them impractical for most needs. Given the justifiably severe practical ethical constraints in medical trials, ISTs have emerged as a potential alternative mechanism to test and optimize innovations prior to or during their use, offering a new way to efficiently qualify and quantify medical technologies, and further personalize their application (Table 1). ISTs have already enabled major breakthroughs across a broad swath of industries including nuclear, automotive, and aviation (4); they seem poised to have even more to offer to the world of human health. Related to artificial intelligence (AI) technologies alone, given the ongoing acceleration of AI innovations in healthcare, in silico technology provides an objective means to enhance their transparency, trustworthiness, generalizability, and interpretability (8, 9).

**Table 1.** IST-DT benefits across the spans of health conditions.

Massive reduction in trials time and cost Assessing specific usefulness of medical products Designing efficient human trials Cost-efficient prototyping Qualifying and validating medicine efficiently Reducing animal trials Driving definitive quantitative endpoints

While the potentials of in silico methods in medicine are intriguing, their realization is far from reality. This is due to a number of obstacles that can be broadly recognized into 5 categories: coordinational, scientific, disseminational, ethical, and economical. Overarching these obstacles is a lack of a coherent goal, or “moonshot,” that is shared and owned across the diversity of stakeholders, so that different progressions can be compelled towards a common objective. A moonshot can be generically defined as an objective that (i) excites and inspires the public, academia, and industry; (ii) helps solve an important societal issue; (iii) is truly disruptive and groundbreaking; (iv) focuses on areas where the underpinning science is at a stage to make a major breakthrough feasible; (v) is specific and well defined in what it sets out to achieve, with a clear time frame for completion; (vi) generates significant additional benefits (10); and (vii) facilitates global harmonization. With the growing evidence of the need for cost-effective, scalable healthcare solutions, and the rising interest in personalized medicine, defining a moonshot is imperative. A moonshot can serve as an overarching goal and a guiding roadmap to align efforts across diverse stakeholders.

## Defining a vision: a DT for every individual

A roundtable of leading thought-leaders, scientists, and regulators from academia, industry, government, and funding agencies was convened in spring 2024 in conjunction with the first International Summit on Virtual Imaging Trials in Medicine (VITM24), to define a common goal for the role of in silico and DT technology in medicine, identify gaps, and prioritize areas for development. The group (whose participants are listed in the Acknowledgments), vested in shaping the future of medicine and motivated by the enormous potential of in silico methods in medicine, engaged in a formative discussion that formed the following moonshot definition:

*Forming and fostering a digital twin of every single human person, integrated into the person's medical record, owned by the person, progressively and continually updated with new data as they become available, used to deliver optimized care for the individual, and used (with the person's permission) for technology assessment, real-world evidence, and population aggregate analysis.*

While ambitious, such a goal is attainable if we consider a characterization of a human DT that is progressive in nature: in the definition of the DT for a young individual or one from whom little medical data are available, the DT would be a rough approximation based on the characteristics of the population within which the individual could be most closely characterized. But as new data become available, the twin progresses to encompass more personalized information. The virtual and the physical realities maintain a bidirectional interaction, reflecting the progressive nature of the twin expressed in the moonshot definition. The twin can then be used as a resource to test and optimize a planned intervention for the care of the individual prior to the actual intervention. Interventions applied to a targeted cohort of DTs would effectually serve as a virtual trial. This approach would be generically applicable across the diversity of health conditions including cancer, cardiovascular disease, diabetes, geriatrics, and obesity.

## Current state of the art and critical science problems

At present, IST and DT technologies in medicine are already advancing the simulation of disease processes, imaging processes, and patient response to treatments. However, their full potential is constrained by several critical gaps and obstacles. As noted previously, these generally fall into the 5 categories of coordinational, scientific, disseminational, ethical, and economical.

On the coordinational front, there is a significant need for clarity of scope. A defined moonshot is not only a foundational step in that direction, but also clarifies the complementary role of regulators, industry, and academia toward the moonshot.

On the scientific front, there is a need to broaden the ongoing work for modeling diverse diseases, the diversity of disease presentations, disease progression and interaction of therapeutic approaches, and continued integration of multiscale physical, chemical, and biological foundations of the human body and maladies. Comprehensive representation of diseases as well as the anatomical variability of humans is integral to this goal. Achieving a functional DT requires advancements in computational modeling, high-fidelity simulation techniques, multiscale physiological modeling, and integration of data-driven machine learning algorithms to process and extract meaningful information from clinical and imaging data. Recent studies have explored these issues, such as credibility assessment frameworks for in silico clinical trials (8) and AI-driven modeling techniques for patient-specific simulations (11).

As it is nearly impossible to fully model the health state with prefect representation, the necessary scientific approximations should be validated for the specific context of use for the in silico models. The scientific development should follow the workflow of design, validation, and implementation toward predicting clinical trial outcomes using DTs. Furthermore, effective data formatting and processing pipelines and AI-driven analytics must be developed to dynamically and bidirectionally update DTs with real-world patient data, ensuring adaptability and accuracy over time. In tandem with scientific development, there is a need for harmonization and standardization to facilitate standardized resources and protocols for the efficient use of such resources. Establishing data and model standards facilitate credibility.

On the dissemination front, there exists a notable deficit in skilled expertise in the IST-DT technologies. As the science and resources of these technologies advance, we need to have adequate plans in place to develop expertise for users and implementers. Graduate and continuing education should include the science and the use of IST-DT resources. Training of physicians should ensure they have access to necessary technology, and comfort and confidence in using them as a complement for clinical evidence. The user engagement can be enhanced by developing prototypes and apps for data collection so that genetic data and technology can be integrated into clinical workflow to automate health index analyses and to improve disease-specific outcomes.

On the ethical front, we to address data access and privacy. Specifically, there is a need to ensure data accessibility while maintaining privacy and security, establishing an economical framework for data access and sharing, and building on mechanisms like the General Data Protection Regulation (12). Related is the importance of patient ownership of DTs, as demonstrated by Germany's model in which patients own their data (13). Patient ownership and engagement in data sharing should recognize the willingness of a person to share information if it is well managed and used toward better outcomes. Further, as in any healthcare resource that is enabled and aimed to serve the public, we need to ensure individualized patient access to technology so all persons can all equitably benefit from the value that the DT technology offers.

On the economic front, there is a need for continued research funding for the effectual development of the IST-DT technologies. For implementation and clinical use, the economics are bound to regulatory confidence in the utility of the technology. There is a need to overcome regulatory hurdles by involving regulators as part of the progression of the technology including ensuring strong engagement of biomedical engineers at the FDA using existing programs such as the Centers of Excellence for Regulatory Science and Innovation (14). Regulatory confidence paves the way toward securing reimbursement from insurance companies for the use of IST-DT–based technologies in clinical practice. Integrating DTs into healthcare and leveraging them for preventive care via insurance companies are pivotal for advancing health equity and personalized medicine. Setting up infrastructure for validation and integrating DTs into general practice, including risk assessment and mitigation, would be integral to this goal.

## Priority areas shaping the path to a new era in healthcare

To advance IST-DT into the future of medicine, stakeholders should be engaged nationally and globally, fostering collaboration and a shared vision. Establishing a digital Contract Research Organization framework is one way to foster systematic processes for the development and execution of ISTs, and crucial community and society engagement for building trust and awareness. Transparent communication and early discussions will drive acceptance, addressing gaps through task-forcing and focused dialogue.

Ensuring sustained progress toward the moonshot prioritizes engagements at multiple levels:

1. Advocating with funding agencies fueling acceleration of scientific development
2. Forming multidisciplinary teams to foster collaboration (e.g. initiating a regulatory science collaborative community)
3. Identifying and engaging standards communities to foster interoperability
4. Harmonizing efforts across regulators and academics towards defining good simulation practices
5. Fostering the uptake of in silico tools by the industry to facilitate professional resource development
6. Developing curricula for workforce trainings to ensure adequate expertise and adaption
7. Engaging with patients and patient advocacy communities to secure public trust and to put the development in the service and discretion of public

The progress toward personalized, data-driven healthcare should be built upon collaboration, innovation, and a shared vision. The path to a DT for every individual represents both a technical and a philosophical shift in medicine. By aligning stakeholders around a common moonshot and addressing the foundational challenges, ISTs and DTs can move toward a future in which patient-specific care is accessible, efficient, and equitable. This goal requires a cross-disciplinary collaboration and continuous dialogue to overcome barriers and ensure the responsible implementation of DT technology.

## Data Availability

There are no data directly underlying this work.
